# Towards optimal model evaluation: enhancing active testing with actively improved estimators

**DOI:** 10.1038/s41598-024-58633-3

**Published:** 2024-05-09

**Authors:** JooChul Lee, Likhitha Kolla, Jinbo Chen

**Affiliations:** Department of Biostatistics, Epidemiology and Informatics, Perelman School of Medicine, University of Pennsylvania, Philadelphia, PA 19104 USA

**Keywords:** Statistics, Applied mathematics, Computer science

## Abstract

With rapid advancements in machine learning and statistical models, ensuring the reliability of these models through accurate evaluation has become imperative. Traditional evaluation methods often rely on fully labeled test data, a requirement that is becoming increasingly impractical due to the growing size of datasets. In this work, we address this issue by extending existing work on active testing (AT) methods which are designed to sequentially sample and label data for evaluating pre-trained models. We propose two novel estimators: the Actively Improved Levelled Unbiased Risk (AILUR) and the Actively Improved Inverse Probability Weighting (AIIPW) estimators which are derived from nonparametric smoothing estimation. In addition, a model recalibration process is designed for the AIIPW estimator to optimize the sampling probability within the AT framework. We evaluate the proposed estimators on four real-world datasets and demonstrate that they consistently outperform existing AT methods. Our study also shows that the proposed methods are robust to changes in subsample sizes, and effective at reducing labeling costs.

## Introduction

Machine learning algorithms and statistical models in recent years have provided innovative solutions across various domains including disease risk prediction and diagnostic image classification^1–3^. Alongside model development, assessment of model performance using test data is essential to foster implementation^4,5^. In real-world settings, model evaluation has traditionally required a fully labeled dataset, overlooking the limiting factors of costs and human effort required for outcome labeling. To circumvent this issue, a small batch of the test dataset can be sampled at the trade-off of reduced statistical power for model evaluation. Hence, statistical methods for model evaluation aimed at improving estimation efficiency with limited labeled data are critical. To this end, we focus on building sampling strategies to sequentially select subsets of data for labeling and developing efficient estimators for model performance metrics.

Recent work has proposed sampling methods to optimally select subsets of data for model evaluation when outcomes are not measured^6,7^. Sawad et al.^6^ proposed sampling distributions minimizing the asymptotic variance of an estimator for $\text {F}$-measures. Kossen et al.^7^ considered sampling distributions based on the cross-entropy loss for classification models and the squared error loss for regression models. Yilmaz et al.^8^ devised sampling distributions minimizing the asymptotic mean squared error of an estimator for predictive accuracy metrics. These methods rely on unknown prediction models that pertains to the main interest of the study. One approach to deal with this limitation is to use pre-trained models as substitutes for the unknown models. In this approach, sampling efficiency can be compromised if the trained models provide less accurate predictions. Active testing^7^ (AT) has been proposed as a promising solution to these challenges. AT is a sampling strategy where subsets of data are selected for labeling in an iterative manner and characteristics of the previously selected subsample inform the selection of the next set.

Here is a general outline of the AT algorithm with a pre-specified initial sampling probability $\pi ^{0}$ to evaluate a trained model $g(\cdot )$. For the *s*th sampling step,

1. assign a sampling probability, $\pi ^{s-1}$, updated at the $(s-1)$th step, to each data point in the remaining unlabeled test data. Then a subsample of subjects is selected and their outcomes are labeled based on $\pi ^{s-1}$.
2. Use the labeled data obtained from the 1st to the *s*th step for calculating the performance metrics to assess $g(\cdot )$.
3. Build an updated version of the model, denoted $g^s(\cdot )$, using the labeled data. Then derive a sampling probability $\pi ^{s}$ based on $g^s(\cdot )$.
4. Continue iterating through the steps until a pre-specified stopping criterion is met.

This AT algorithm allows the selection of informative subsets of data to efficiently estimate model performance metrics, compared with random selection of subsets^7^. In this algorithm, the performance of AT depends on two key factors: the estimation approach for the performance metrics in the second step and the updating approach for the sampling probability in the third step.

**Figure 1:**
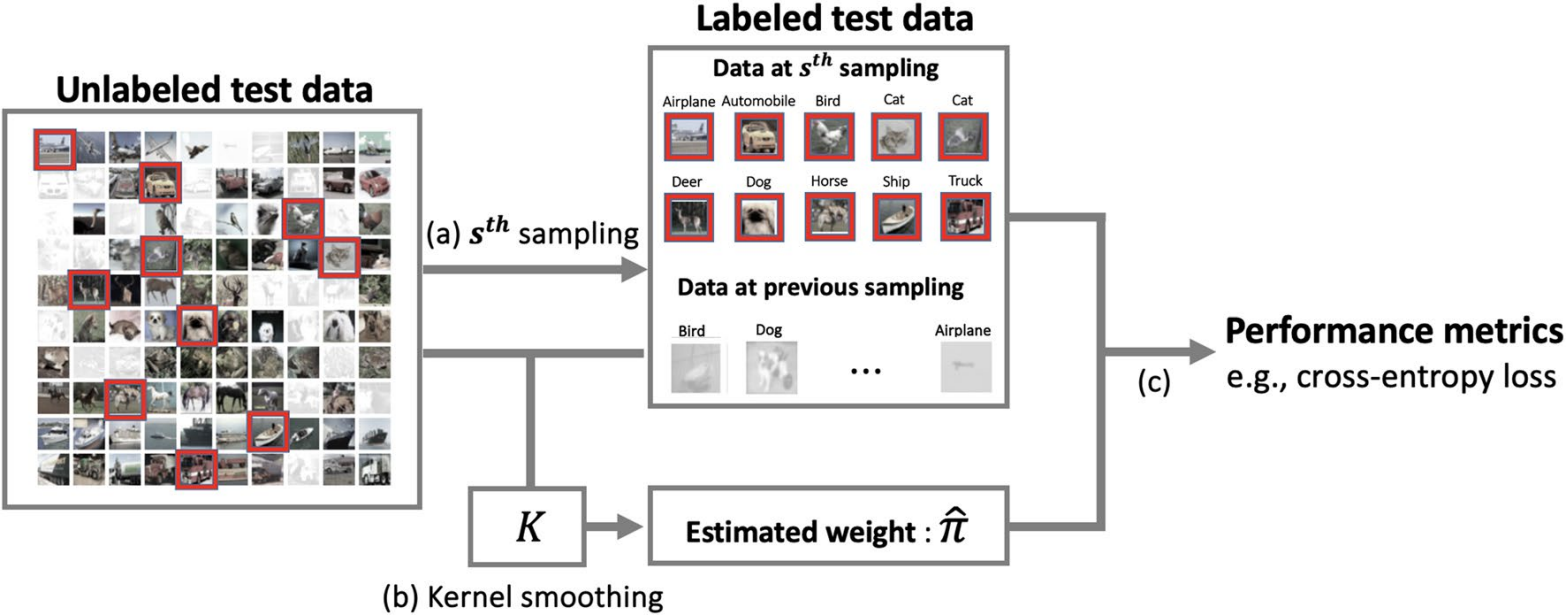
Overview of the proposed active model evaluation. For the *s*th sampling step, (**a**) select a subsample among the remaining unlabeled data to label their outcomes. (**b**) Conduct the kernel smoothing estimation to obtain estimated weights based on the remaining unlabeled data and the labeled data accumulated from the 1st to the *s*th sampling step. (**c**) Compute model performance metrics using the estimated weights and the accumulated labeled data.

In AT, the distribution of the labeled data differs from that of the unlabeled data due to the selective sampling. This sampling bias leads to biased estimation of the model performance metrics. To remove this bias, Farquhar et al.^9^ proposed the levelled unbiased risk (LUR) estimator for learning with a general form of loss functions under sequential selective labeling. Kossen et al.^7^ adopted the LUR estimator for estimating model performance metrics in AT. This method employs adjusted inverse probability weightings (IPW) during sequential sampling steps to counteract the statistical bias in the target function.

Motivated by the missing data literature where replacing true sampling weights by well-estimated weights can improve estimation efficiency of IPW estimators^10,11^, we propose two actively improved (AI) estimators for model performance metrics in this work. These estimators are developed using estimated weights from nonparametric smoothing estimation (see Fig. 1). Our contributions in this paper are as follows:

1. We propose the AI levelled unbiased risk (AILUR) estimator, which is constructed by replacing the true sampling weights in the LUR estimator with estimated weights. The second proposed method, the AI inverse probability weight (AIIPW) estimator, is constructed based on the estimated probability that a subject is ever included in the labeled data throughout the sampling steps. Compared with the AILUR method, AIIPW is memory-efficient since it does not need to store the true sampling weights throughout the labeling process, as long as the sampling probabilities are a function of $g(\cdot )$.
2. To maximize the benefit of AT, we propose a model re-calibration process to update the sampling probability, tailored for the AIIPW estimator (see the “Methods” section). Based on the proposed estimators for model performance metrics and this re-calibration process, we propose a practical AT algorithm. We empirically demonstrate that the proposed algorithm outperforms the existing AT method across various real-world datasets. Moreover, we show that it is robust to variations in subsample size and the number of sampling steps.

## Methods

### Fashion MNIST data

The Fashion-MNIST dataset is a collection of 70,000 grayscale images, each measuring 28 $\times$ 28 pixels. The images are divided into ten unique categories, such as ‘shirt’ and ‘bag’, with each category containing 7000 images. This balance is crucial for fair machine learning training. The dataset is split into 60,000 training data and 10,000 testing data. The Fashion MNIST dataset is available at https://github.com/zalandoresearch/fashion-mnist. To mimic model training outlined in Kossen et al.^7^, we selected 250 data points from the training data of 60,000 using stratified sampling to obtain equal sizes across 10 categories. We built a ResNet-18 model to classify categories of clothing using the training data of size 250. Then we evaluated its performance using the test data.

### CIFAR-10 data

The CIFAR-10 dataset is a collection of 60,000 color images, each measuring 32$\times$32 pixels. These images are evenly distributed across ten categories such as ‘airplane’ and ‘dog’, with 6000 images in each. The dataset is partitioned into two sets: 50,000 images for training and 10,000 images designated for testing. The CIFAR-10 dataset can be accessed at https://www.cs.toronto.edu/%7Ekriz/cifar.html. A ResNet-18 model for image classification was trained using the training data and then its performance was assessed on the test data.

### Drug consumption data

The drug consumption dataset holds information on 1,885 respondents, each characterized by 12 attributes. These include personality measures for neuroticism, extraversion, agreeableness, impulsivity, and sensation seeking. Other demographic details provided are education level, age, gender, country of residence, and ethnicity. Participants also provided information on their consumption of 18 drugs, both legal and illegal, such as alcohol, cannabis, and cocaine. Notably, a fictitious drug named ‘Semeron’ was added to spot those exaggerating their drug use. For each drug, there are seven possible usage labels, ranging from ‘Never Used’ to ‘Used in Last Day’. We used 12 attributes as predictors and usage of the Semeron drug as the outcome. We randomly selected 150 data points to build a random forest classifier for the usage of the Semeron drug, and used the remaining data as test data for model evaluation. The dataset can be accessed freely at https://archive.ics.uci.edu/dataset/373/drug+consumption+quantified.

### Non-alcoholic fatty liver disease (NAFLD) data

The non-alcoholic fatty liver disease (NAFLD) dataset is composed of EHR from patients seen at the University of Pennsylvania. 300 patients and 635 patients were identified as biopsy-confirmed and imaging-confirmed NAFLD cases, respectively. 2805 patients who were not diagnosed by NAFLD were derived from the Penn Medicine Biobank database as control subjects. 41 variables, including demographics, laboratory measurements, and medication were considered as predictors in the model. For missing value imputation, we used the mean for continuous variables, and the mode for categorical variables. To consider the real world setting where the distribution of test data differs from one of the training data, we created two datasets: a training dataset with EHR records from 300 biopsy-confirmed NAFLD cases and 1000 controls; and a testing dataset with EHR records from 635 imaging-identified cases and the remaining 1905 controls. We built a random forest classifier for NAFLD using the training dataset and validate it’s performance using the testing dataset.

### Trained models to be assessed

For the Fashion-MNIST and CIFAR-10 datasets, we normalized the pixel values on each training image to a range between 0 and 1, and built the ResNet-18 models using the codes shared in Kossen et al.^7^. We employed a stochastic gradient descent optimizer with a learning rate of 0.1, weight decay of 5 $\times$ $10^{-4}$, and momentum of 0.9. The batch size was set to 128, and we used a cosine annealing schedule for the learning rate. For Drug consumption data and NAFLD, we trained random forest classifiers using the ‘randomForest’ package^12^ in the R programming language. We treated the training datasets as inaccessible data during model evaluation and considered the test datasets as unlabeled data.

### Problem setup and LUR estimator

Let *Y* be a outcome variable and $\textbf{X}$ be the *p* dimensional vector of covariates. Let $g(\textbf{X})$ denote a model trained by an external data. In this work, we aim to evaluate the performance of the model $g(\textbf{X})$ using a loss function of model performance metrics,

1 $$\begin{aligned} \mathscr {M} \equiv \textbf{E}[\mathscr {L}\{g(\textbf{X}),Y\}]. \end{aligned}$$

Depending on the definition of $\mathscr {L}(\cdot )$, the quantity $\mathscr {M}$ can represent performance metrics such as the mean squared error, cross-entropy, and Akaike information criterion. We assume that a test data for the model evaluation includes only observations of $\textbf{X}$, $\{\textbf{X}_i, i = 1,...,N\}$ where $\textbf{X}_i$’s are independent and identically distributed. Data for *Y*, however, is not available. Since it is infeasible to annotate all outcomes in the test data for large *N*, AT selects a subset of the test data to label the outcomes.

In the active testing algorithm, the already labeled subsample in the previous steps can be treated as sampled with probability 1 for the next sampling steps since it, along with the newly labeled subsample, is utilized for model validation. Let $\delta ^s_i$ indicate whether the *i*th data point is selected for labeling at the *s*th sampling or not ($\delta ^s_i = 1$ : labeled; $\delta ^s_i = 0$ : unlabeled). Let $\pi {\{g^s(\textbf{X}_i), \delta ^{s-1}_i\}}\equiv \delta ^{s-1}_i + (1-\delta ^{s-1}_i)P\{\delta ^s_i =1 | g^s(\textbf{X}_i) \}$ denote the sampling probability for the $i^{th}$ patient where $0 < \pi {\{g^s(\textbf{X}_i), \delta ^{s-1}_i\}} \le 1$. If the *i*th data point is selected in the previous steps, $\pi \{g^s(\textbf{X}_i), \delta ^{s-1}_i = 1\} = 1$ as it should be automatically included in the labeled test data. We therefore can include labeled data points from the 1st to the $(s-1)$th sampling into the labeled test data at the *s*th sampling step. If the *i*th data point is in the remaining unlabeled test data (i.e., $\delta ^{s-1}_i = 0$), we perform sampling with the sampling probability $P\{\delta ^s_i =1 | g^s(\textbf{X}_i) \}$ under Poisson sampling scheme. Let $n^*_s$ be the newly labeled data size at the *s*th sampling from the remaining unlabeled data. Then, the cumulative labeled data size after performing the *s*th sampling is $\sum ^N_{i=1}\delta ^s_i = \sum ^N_{i=1}\delta ^{s-1}_i + n^*_s$. The labeled test data would consist of the already labeled data from the previous sampling steps and newly labeled data at the *s*th sampling step. Under Poisson sampling scheme, labeled data size $n^*_s$ is random with $\textbf{E}(n^*_s) = n_s$. The data available after the *s*th sampling can be represented as $\{\delta ^s_i, \delta ^s_iY_i, \textbf{X}_i, i = 1,...,N\}$.

In the AT setting, the distribution of labeled data is not the same as that of the unlabeled data due to selective labeling. To remove this sampling bias, the LUR estimator^9^ at the *s*th step is an existing method to achieve the unbiased estimation of $\mathscr {M}$ using the labeled data,

2 $$\begin{aligned} \widehat{\mathscr {M}}^{\text {LUR}}_s = \frac{1}{sN}\sum ^{s}_{j=1}w_j\sum ^{N}_{i=1}\frac{\delta ^{j}_i\mathscr {L}\{g(\textbf{X}_i),Y_i\}}{\pi {\{g^j(\textbf{X}_i), \delta ^{j-1}_i\}}}, \end{aligned}$$

where the weight $w_j = N(N-s)/\{(N-j)(N-j+1)\}$ is adjusted in each sampling to remove the statistical bias for $\mathscr {M}$. However, such estimators based on true inverse probability weighting (IPW) may be inefficient since the estimation variability for $\mathscr {M}$ can be inflated if data points are selected with small sampling probabilities^13^.

### AILUR estimator

Recall that the sampling probability at the *s*th sampling step is $\pi {\{g^s(\textbf{X}_i), \delta ^{s-1}_i\}} = \delta ^{s-1}_i + (1-\delta ^{s-1}_i)P\{\delta ^s_i =1 | g^s(\textbf{X}_i) \}$. We know that $\pi {\{g^s(\textbf{X}_i), \delta ^{s-1}_i= 0\}} = P\{\delta ^s_i =1 | g^s(\textbf{X}_i) \}$ is the sampling probability at the *s*th sampling used for acquiring the new labeled data from the remaining unlabeled test data. For building the AILUR estimator, we focus on estimating $P\{\delta ^s_i =1 | g^s(\textbf{X}_i)\}$. Using the introduced notation, we can express the new labeled data as $\{\delta ^{s}_i(1-\delta ^{s-1}_i)(\textbf{X}_i, Y_i), \delta ^{s}_i, i = 1,...,N\}$, and the remaining unlabeled test data as $\{(1-\delta ^{s-1}_i)\textbf{X}_i, i = 1,...,N\}$. We consider an kernel smoothing estimator for $P\{\delta ^s_i =1 | g^s(\textbf{X}_i)\}$,

$$\begin{aligned} \hat{P}\{\delta ^s_i =1 | g^s(\textbf{X}_i)=z\} =\frac{\sum ^{N}_{i=1}\delta ^s_i(1-\delta ^{s-1}_i) K_b\{g^s(\textbf{X}_i)-z\}}{\sum ^{N}_{i=1}(1-\delta ^{s-1}_i)K_b\{g^s(\textbf{X}_i)-z\}} \end{aligned}$$

where $K_b$ is a kernel function with a bandwidth *b*. Let $\hat{\pi }{\{g^s(\textbf{X}_i), \delta ^{j-1}_i\}} = \delta ^{s-1}_i + (1-\delta ^{s-1}_i)\hat{P}\{\delta ^s_i =1 | g^s(\textbf{X}_i)\}$ be the estimator for $\pi {\{g^s(\textbf{X}_i), \delta ^{s-1}_i\}}$. By replacing $\pi (\cdot )$ in the Eq. (2) with $\hat{\pi }(\cdot )$, we propose the AILUR estimator of ${\mathscr {M}}$ at the *s*th step,

3 $$\begin{aligned} \widehat{\mathscr {M}}^{\text {AILUR}}_s = \frac{1}{sN}\sum ^{s}_{j=1}w_j\sum ^{N}_{i=1}\frac{\delta ^{j}_i\mathscr {L}\{g(\textbf{X}_i),Y_i\}}{\hat{\pi }{\{g^j(\textbf{X}_i), \delta ^{j-1}_i\}}}, \end{aligned}$$

By the property of nonparametric kernel smoothing estimation for $P\{\delta ^s_i =1 | g^s(\textbf{X}_i)\}$, we have that the difference between $\pi (\cdot )$ and $\hat{\pi }(\cdot )$ can be very small under some regularity assumptions. We hence show that $\widehat{\mathscr {M}}^{\text {AILUR}}$ is an asymptotically unbiased estimator of $\mathscr {M}$.

### AIIPW estimator

One challenge to construct the estimators in the Eqs. (2) or (3) requires the information of all true sampling weights (or estimated weights) used throughout the sampling, which increases memory costs. We propose the AIIPW estimator to alleviate the problem. To build the estimator, we first investigate the conditional expectation of the indicator on $\textbf{X}$, $\textbf{E}\{\delta ^s_i|g(\textbf{X})\}$. By iterative double expectation, we have

$$\begin{aligned} \textbf{E}\{\delta ^s_i|g(\textbf{X})\}&= {\left\{ \begin{array}{ll} P\{\delta ^1_i =1 | g(\textbf{X})\}, &{} \text {if s= 1},\\ \textbf{E}\{\delta ^{s-1}_i|g(\textbf{X})\} + P\{\delta ^s_i =1 | g(\textbf{X})\}\prod ^{s-1}_{j=1}[1-P\{\delta ^j_i =1 | g(\textbf{X})\}], &{} \text {if s > 1}. \end{array}\right. } \end{aligned}$$

Using the true weight, we can construct an IPW estimator of ${\mathscr {M}}$,

4 $$\begin{aligned} \widetilde{\mathscr {M}}^{\text {IPW}} = \frac{1}{N}\sum ^{N}_{i=1}\frac{\delta ^{s}_i\mathscr {L}\{g(\textbf{X}_i), Y_i\}}{\textbf{E}\{\delta ^s_i|g(\textbf{X})\}}. \end{aligned}$$

However, the estimator still depends on historical sampling probabilities. To avoid this, we construct an kernel smoothing estimator to replace $\textbf{E}\{\delta ^s_i|g(\textbf{X})\}$,

5 $$\begin{aligned} \hat{\textbf{E}}\{\delta ^s_i|g(\textbf{X}_i)=z\} =\frac{\sum ^{N}_{i=1}\delta ^s_i K_b\{g(\textbf{X}_i)-z\}}{\sum ^{N}_{i=1}K_b\{g(\textbf{X}_i)-z\}} \end{aligned}$$

By replacing $\hat{\textbf{E}}\{\delta ^s_i|g(\textbf{X})\}$ in the Eq. (4) with $\hat{\textbf{E}}\{\delta ^s_i|g(\textbf{X})\}$, we propose the AIIPW estimator of ${\mathscr {M}}$at the *s*th step,

6 $$\begin{aligned} \widehat{\mathscr {M}}^{\text {AIIPW}}_s = \frac{1}{N}\sum ^{N}_{i=1}\frac{\delta ^{s}_i\mathscr {L}\{g(\textbf{X}_i),Y_i\}}{\hat{\textbf{E}}\{\delta ^s_i|g(\textbf{X}_i)\}}. \end{aligned}$$

AIIPW for ${\mathscr {M}}$ is memory-efficient since the estimator at the $s^{th}$ step does not rely on all true sampling weights (or estimated weights) used throughout the first step to the *s*th step, $\pi {\{g^j(\textbf{X}_i), \delta ^{j-1}_i\}}$ (or $\hat{\pi }{\{g^j(\textbf{X}_i), \delta ^{j-1}_i\}}$) for $j=1,...,s$.

**Algorithm 1:**
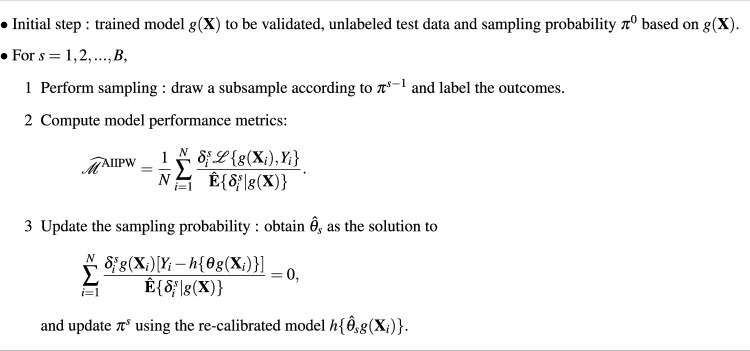
Actively improved model evaluation algorithm.

### Active model evaluation algorithm

Specifying the sampling probability in the active testing is important to acquire more informative labeled data for improvement of estimation efficiency. Using expected true loss conditional on $\textbf{X}$ is one approach for driving sampling probabilities when the outcome is unlabeled, $P\{\delta ^s =1 | \textbf{X}\} \propto \textbf{E}[\mathscr {L}\{g(\textbf{X}),Y\}|\textbf{X}]$^7^. The expected loss is larger, the corresponding data points are more likely to be selected for labeling. For example, for binary classification models, we can consider the expected cross-entropy function conditional on $\textbf{X}$,

$$\begin{aligned} P(\delta ^s =1 | \textbf{X}) \propto P(Y=1|\textbf{X})\log {g(\textbf{X})} + \{1-P(Y=1|\textbf{X})\}\log {\{1-g(\textbf{X})\}}. \end{aligned}$$

By replacing the true $P(Y=1|\textbf{X})$ with $g(\cdot )$, the sampling probability can be approximated. Since the performance of sampling depends on prediction accuracy of the trained model, we consider model re-calibration with the labeled data $\{\delta ^s_i, \delta ^s_iY_i, \textbf{X}_i, i = 1,...,N\}$ using the estimating equation

7 $$\begin{aligned} \sum ^{N}_{i=1}\frac{\delta ^s_ig(\textbf{X}_i)[Y_i- h\{\theta g(\textbf{X}_i)\}]}{\hat{\textbf{E}}\{\delta ^s_i|g(\textbf{X})\}} = 0, \end{aligned}$$

where $h(\cdot )$ is an arbitrary function (e.g., $h = \{1+\exp (-\theta g(\textbf{X}_i)\})\}^{-1}$ for a logistic regression and $h = \theta g(\textbf{X}_i)\}$ for a linear regression). We can also conduct re-calibration of multinomial logistic regressions in form of the Eq. (7) (see Appendix). Let $\hat{\theta }_s$ be the solution of the Eq. (7) and $h\{\hat{\theta }_s g(\textbf{X}_i)\}$ be the re-calibrated model. Based on the re-calibrated model $h\{\hat{\theta }_s g(\textbf{X}_i)\}$, we update the sampling probability. Algorithm 1 provides a summary of our general framework.

### Extension to predictive accuracy metrics

We now focus on evaluating classification models using a general form of predictive accuracy metrics,

8 $$\begin{aligned} \mathscr {D} \equiv \frac{\textbf{E}[d_{1}\{g(\textbf{X}),Y\}]}{\textbf{E}[d_{2}\{g(\textbf{X}),Y\}]}. \end{aligned}$$

The quantity $\mathscr {D}$ is varied depending on the definition of $d_1$ and $d_2$ such as the true positive rate, false positive rate, positive predictive value, and $\text {F}_1$ score (see Table 1).

Based on the labeled data $\{\delta ^s_i, \delta ^s_iY_i, \textbf{X}_i, i = 1,...,N\}$ and the estimated weights from the Eq. (5) after labeling at the *s*th sampling, we can construct the AIIPW estimator of the metrics ${\mathscr {D}}$,

9 $$\begin{aligned} \widehat{\mathscr {D}}^{\text {AIIPW}}_s = \frac{\sum ^{N}_{i=1}\delta ^{s}_im_{1}\{g(\textbf{X}_{i}),Y_{i}\}/\hat{\textbf{E}}\{\delta ^s_i|g(\textbf{X})\}}{\sum ^{n}_{i=1}\delta ^{s}_im_{2}\{g(\textbf{X}_{i}),Y_{i}\}/\hat{\textbf{E}}\{\delta ^s_i|g(\textbf{X})\}}. \end{aligned}$$

For example, the AIIPW estimator for the true positive rate from the Eq. (9) can be presented as

$$\begin{aligned} \widehat{\text {TPR}}^{\text {AIIPW}}_s = \frac{\sum ^{N}_{i=1}\delta ^{s}_i\textbf{I}\{g(\textbf{X}) > c\}Y_{i}/\hat{\textbf{E}}\{\delta ^s_i|g(\textbf{X})\}}{\sum ^{N}_{i=1}\delta ^{s}_iY_{i}/\hat{\textbf{E}}\{\delta ^s_i|g(\textbf{X})\}}, \end{aligned}$$

where *c* is the risk cutoff.

**Table 1:** Examples for representative predictive accuracy metrics at a risk cutoff *c*.

Measure	$d_1(z_1, z_2)$	$d_2(z_1, z_2)$
True positive rate	$\textbf{I}(z_1>c)z_2$	$z_2$
False positive rate	$\textbf{I}(z_1>c)(1-z_2)$	$(1-z_2)$
Positive predictive value	$\textbf{I}(z_1>c)z_2$	$\textbf{I}(z_1>c)$
Negative predictive value	$\textbf{I}(z_1<c)(1-z_2)$	$\textbf{I}(z_1<c)$
$\text {F}_1$ score	$\textbf{I}(z_1>c)z_2$	$\textbf{I}(z_1>c) + z_2$

**Table 2:** Names and details for the combinations of the methods used in the numerical studies.

Method	Estimator for performance metrics	Model used for sampling probability	Figure
LUR-Ori	LUR estimator	Original model $g(\cdot )$	Figures 3, 5 and 6
LUR-Rec	LUR estimator	Re-calibrated model	Figures 5 and 6
LUR-RF	LUR estimator	Random forest	Figures 5 and 6
AILUR-Ori	AILUR estimator	Original model $g(\cdot )$	Figures 3, 4 and 5
AILUR-RF	AILUR estimator	Random forest	Figures 3, 4 and 5
AILUR-Rec	AILUR estimator	Re-calibrated model	Figures 2, 3, 4 and 5
AIIPW-Ori	AIIPW estimator	Original model $g(\cdot )$	Figures 4, 5 and 6
AIIPW-Rec	AIIPW estimator	Re-calibrated model	Figures 2, 3, 4, 5 and 6

LUR, AILUR and AIIPW refer to the levelled unbiased risk estimator, the actively improved levelled unbiased risk estimator, and the actively improved inverse probability weighting estimator, respectively. Ori, RF, and Rec denote the use of the original model, the random forest models, and the proposed re-calibrated models, respectively, when updating the sampling probability.

## Results

We evaluated the efficiency of the AILUR and AIIPW estimators and the proposed method for updating sampling probabilities using four different datasets: Fashion-MNIST^14^, CIFAR-10^15^, Drug Consumption^16^, and Non-alcoholic fatty liver disease (NAFLD) from the Penn Medicine Biobank database. Kossen et al.^7^ used ResNet-18 models developed with the Fashion-MNIST and CIFAR-10 datasets to investigate the performance of the LUR estimator within the AT framework. To compare our proposed methods with LUR under a similar setup, we utilized these models for both the Fashion-MNIST and CIFAR-10 datasets. Fehrman et al.^16^ and Wu et al.^17^ considered random forest models for the prediction of drug consumption and fatty liver disease, respectively. We selected these models to evaluate their performance using the Drug Consumption and NAFLD datasets.

Entropy sampling, an ad-hoc sampling strategy based on the cross-entropy function, is generally competitive with other sampling strategies. In this paper, we consider expected cross-entropy function^7^ conditional on $\textbf{X}$ as the sampling probability at each sampling step for binary outcomes,

10 $$\begin{aligned} P(\delta ^s =1 | \textbf{X}) \propto P(Y=1|\textbf{X})\log {g(\textbf{X})} + \{1-P(Y=1|\textbf{X})\}\log {\{1-g(\textbf{X})\}}, \end{aligned}$$

and for multi-class outcomes,

11 $$\begin{aligned} P(\delta ^s =1 |\textbf{X}) \propto \sum ^{C}_{c=1} P(Y=c|\textbf{X})\log {\{g_c(\textbf{X})\}}, \end{aligned}$$

where *C* is the number of classes in *Y*, $\textbf{X}$ is a vector of predictors, and $g(\cdot )$ and $g_c(\cdot )$ are trained models to be evaluated. We need to consider a replacement, $g^s(\cdot )$, for replacing the true probabilities $P(Y=1|\textbf{X})$ in the equation (10) (or $P(Y=c|\textbf{X})$ in the equation (11)) at the $s^{th}$ sampling step. We consider three different alternatives: the original model $g(\cdot )$ (“Ori”) (or $g_c(\cdot )$ for multi-class classification model), random forest models (“RF”), and the proposed re-calibrated models (“Rec”). In addition, we provide results under the uniform sampling probability (“Uniform”). We considered AIIPW with Ori and Rec since the sampling probability in these schemes is derived as a function of the trained model $g(\cdot )$(see more details in Methods section). The names and details for the combinations of methods are described in Table 2. Details on experiments and alternative sampling functions for the Eqs. (10) and  (11) are provided in the Appendix.

### Comparison of efficiency for the proposed estimators and methods for updating sampling probability

**Figure 2:**
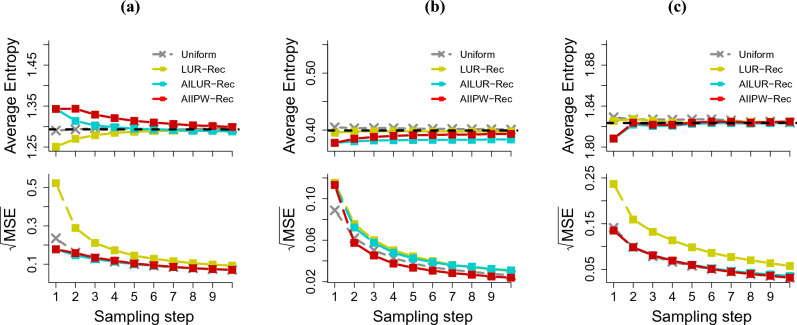
The averaged cross-entropy loss and the averaged square root of the mean squared error (MSE) using (**a**) Fashion-MNIST, (**b**) CIFAR-10, and (**c**) Drug consumption datasets. The estimates at each sampling step were calculated using the accumulated labeled data.

To assess the efficiency of the proposed estimator using the alternative sampling function based on Rec, we first compared AILUR-Rec and AIIPW-Rec with LUR-Rec. We considered 10 sampling steps, a subsample size of 100 at each step, and 1000 repetitions. Estimates from the full test data were set as the benchmark metrics to compare against. Figure 2 displays the average of the cross-entropy loss and the average of the square root of the mean squared error (MSE) for the cross-entropy loss on Fashion-MNIST, CIFAR-10 and Drug Consumption datasets.

As more labeled data accumulated, the cross-entropy loss estimates for all methods approached the benchmark values. AIIPW-Rec consistently outperformed LUR-Rec across every sampling step for all datasets with respect to MSE. In the initial step, the MSEs for AIIPW-Rec were 0.178 for Fashion-MNIST, 0.113 for CIFAR-10, and 0.135 for Drug Consumption, compared to LUR’s MSEs of 0.523, 0.115, and 0.237, respectively. After the tenth sampling step, AIIPW-Rec’s MSEs for Fashion-MNIST, CIFAR-10, and Drug Consumption reduced to 0.070, 0.024, and 0.032 respectively, compared to LUR-Rec’s MSEs of 0.092, 0.030, and 0.057. AILUR-Rec provided similar MSE values to LUR-Rec for CIFAR-10, while it performed better than LUR-Rec for Fashion-MNIST and Drug Consumption, showing performance comparable to AIIPW-Rec.

**Figure 3:**
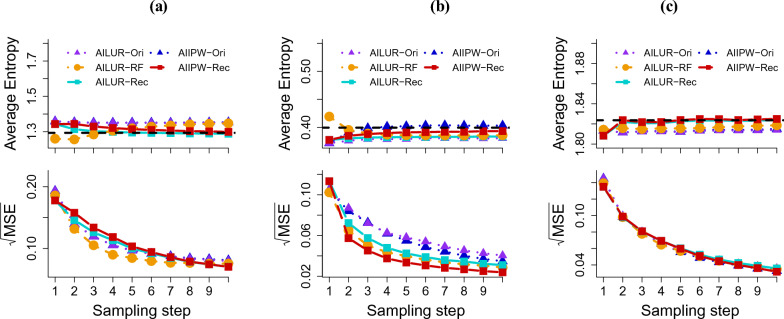
The averaged cross-entropy loss and the averaged square root of the mean squared error (MSE) using (**a**) Fashion-MNIST, (**b**) CIFAR-10, and (**c**) drug consumption datasets. The estimates at each sampling step were calculated using the accumulated labeled data.

Next, we investigated the efficiency of three approaches (Ori, RF, and Rec) for updating the sampling probability. The MSE results are shown in Fig. 3. Applying AILUR-RF to the Fashion-MNIST dataset yielded decreasing MSEs of 0.186 at the first sampling step, 0.084 at the fifth step, and 0.076 at the tenth step. This trend was closely followed when AIIPW-Ori was applied to the Fashion-MNIST dataset, with the MSEs of 0.193 at the first step, 0.097 at the fifth step, and 0.081 at the tenth step. AIIPW-Rec fell behind from the second to the sixth sampling step, but it achieved the lowest MSE at the tenth step (0.178 at the first, 0.103 at the fifth and 0.070 at the tenth). When the proposed estimators were applied to the CIFAR-10 dataset, AILUR-RF provided the MSE (0.102) at the first sampling step, which is similar to that of AIIPW-Ori (0.105) and lower than that of AIIPW-Rec (0.113). From the second sampling step and onwards, AIIPW-Rec yielded the lowest MSEs, compared with AIIPW-Ori and AILUR-RF. The MSEs for AIIPW-Rec at the tenth sampling step was 0.027, trailed by the AILUR-RF’s value of 0.032, and the AIIPW-Ori’s value of 0.040. AIIPW-Rec maintained the superiority with the lowest MSE of 0.024 at the last sampling step, with AILUR-RF’s 0.029 and AIIPW-Ori’s 0.035. For the Fashion-MNIST and CIFAR-10 datasets, AILUR-RF demonstrated better performance than both AILUR-Ori and AILUR-Rec. All methods applied to the Drug Consumption dataset showed comparable MSE trends across the sampling steps, with the MSEs of 0.135, 0.139, and 0.145 at the first sampling step; 0.060, 0.057, and 0.056 at the fifth sampling step; and 0.032, 0.035, and 0.033 at the tenth step for AIIPW-Rec, AILUR-RF, and AIIPW-Ori, respectively. For the Drug Consumption dataset, Ori, RF, and Rec, when applied with the AILUR method, exhibited comparable MSE trends across the sampling steps.

### Extension of the proposed estimators to predictive accuracy metrics and comparison of their efficiency

**Figure 4:**
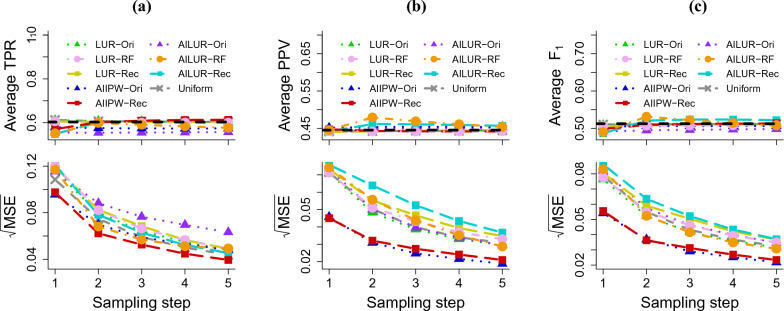
The averaged true positive rate (TPR), positive predictive value (PPV), and $\text {F}_1$ score ($\text {F}_1$), and the averaged square root of the mean squared error (MSE) at risk cutoffs corresponding to false positive rate 0.25 using NAFLD data. The dashed black lines in the top panel are benchmarks calculated using the fully labeled test data (0.600 for TPR, 0.450 for PPV, 0.500 for $\text {F}_1$.

We applied our proposed methods to estimate common predictive accuracy metrics, including true positive rate (TPR), positive predictive value (PPV), and $\text {F}_1$ score. We then assessed their estimation efficiency for the predictive accuracy metrics, considering different alternative sampling functions. Using the NAFLD dataset, We considered a total of five sampling steps, each with a subsample size of 100. The corresponding estimates derived from the full test data served as benchmark values against (TPR: 0.600, PPV: 0.450, $\text {F}_1$: 0.500). Figure 4 presents the averages and MSEs of TPR, PPV, and $\text {F}_1$ at risk cutoffs corresponding to the false positive rate of 0.25. All methods produced accuracy metric estimates close to benchmark values. Across all sampling steps, datasets, and performance metrics, AIIPW and AILUR yielded lower MSEs compared with LUR, with only exception being for the estimation of PPV when the sampling probability was updated based on RF (Fig. 4). AIIPW-Rec outperformed LUR-Rec for all metrics, achieving the MSEs of 0.1 for TPR, 0.045 for PPV, and 0.055 for $\text {F}_1$ in the first sampling step, compared with LUR-Rec’s corresponding values of 0.12, 0.07, and 0.08, respectively. This trend continued into the last sampling step with MSEs of 0.04 for TPR, 0.02 for PPV, and 0.02 for $\text {F}_1$, as opposed to 0.05, 0.04, and 0.04, respectively, for LUR-Rec. In general, AIIPW-Rec showed superior performance for TPR, while both AIIPW-Rec and AIIPW-Ori outperformed other methods for PPV and $\text {F}_1$ in terms of MSE.

**Figure 5:**
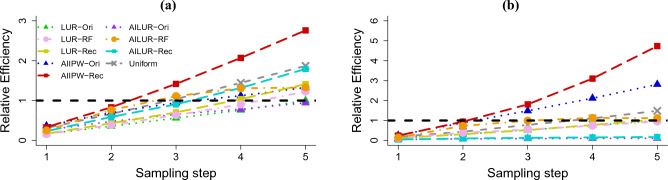
Relative efficiency (RE) for the area under the receiver operating characteristic curve (AUC) and the area under the precision-recall curve (AUPRC) using NAFLD data. (**a**) Relative efficiency for AUC of different methods compared with LUR-Ori. (**b**) Relative efficiency for AUPRC of different methods compared with LUR-Ori.

We next examined the relative efficiency (“RE”) among the estimators and the updating methods for sampling probability. Let $\text {MSE}_{\text {med},s}$ denote MSE for one of the seven combinations of methods {LUR-Ori, LUR-RF, LUR-Rec, Uniform, AIIPW-Ori, AILUR-RF, AIIPW-Rec} at the $s^{th}$ step. We assessed the estimation efficiency of the proposed methods across different sampling steps relative to LUR-Ori at the fifth step, defined as $\text {MSE}_{\text {med},s}/\text {MSE}_{\text {LUR-Ori},5}$. For instance, RE of AIIPW-Rec at the third sampling step compared to LUR-Ori at the fifth sampling step is computed as $\text {MSE}_{\text {AIIPW-Rec},3}/\text {MSE}_{\text {LUR-Ori},5}$. Fig. 5a shows RE for the area under the receiver operating characteristic curve (AUC) among the seven different methods across five sampling steps. The RE values of AIIPW-Rec and AILUR-RF relative to LUR-Ori at the fifth step exceeded one beginning at the third sampling step, which requires only 300 data points given 100 points sampled per step. This indicates that AIIPW-Rec and AILUR-RF can achieve the MSEs comparable to LUR-Ori at the fifth sampling step while saving the labeling cost for more than 200 data points (since we considered a subsample size of 100 in each step). Additionally, the RE values of AIIPW-Ori and LUR-Rec relative to LUR-Ori at the fifth step surpassed one by the fourth batch, and reached 1.234 and 1.518, respectively, by the fifth batch. Uniform sampling also outperformed LUR-Ori with the RE value close to 2 by the fifth batch.

Figure 5b presents RE for the area under the precision-recall curve (AUPRC). AUPRC represents a curve that plots PPV (*y*-axis) against TPR (*x*-axis) across all risk cutoffs. The RE value for AIIPW-Rec relative to LUR-Ori at the fifth step surpassed one by the second sampling step and increased to 5.369 by the fifth batch. Both AIIPW-Ori and AIIPW-RF crossed the RE value of one at the third batch and attained the RE values of 2.986 and 1.342, respectively, by the fifth step. The RE values for LUR-RF and Uniform relative to LUR-Ori at the fifth step started to exceed one starting at the fourth sampling step and reached the RE values of 1.341 and 1.981, respectively, by the last step.

### Effects of subsample size on estimation for performance metrics

**Figure 6:**
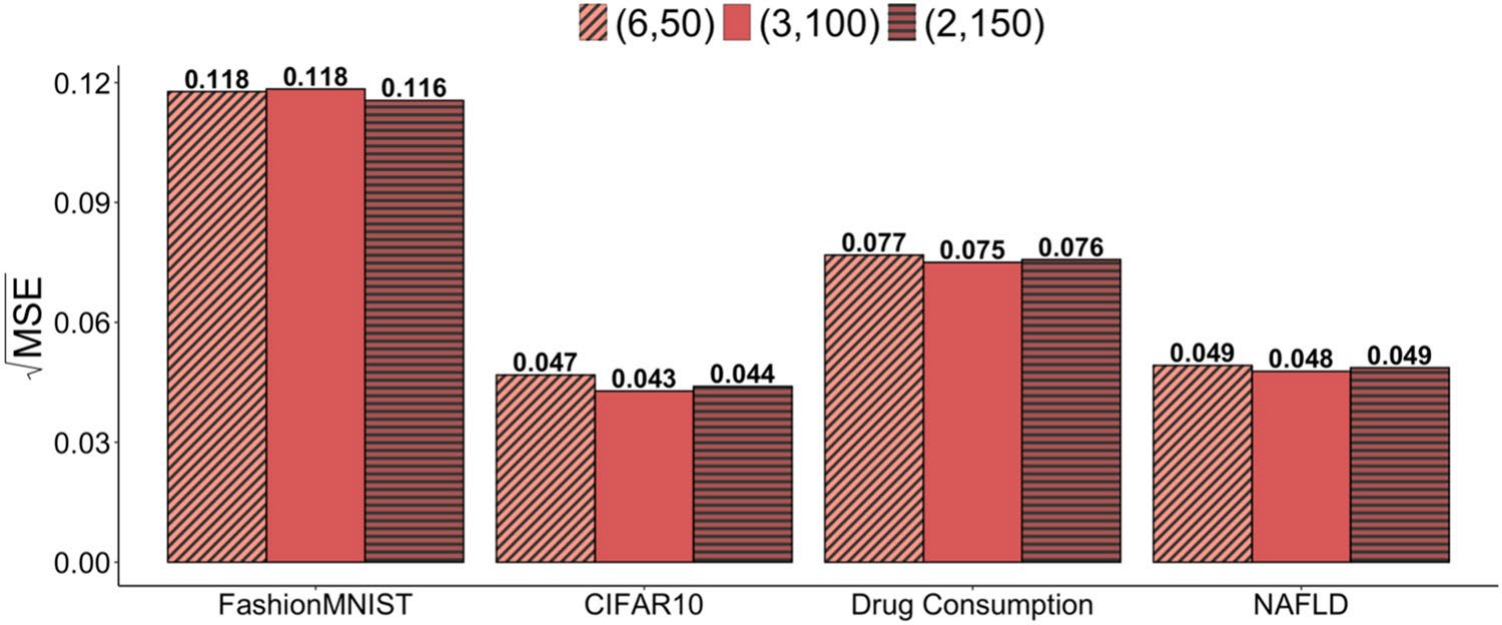
The averaged square root of the mean squared error (MSE) for the cross-entropy loss using fashion-MNIST, CIFAR-10, drug consumption, and NAFLD data. The expected total labeled data size is fixed at 300, and three scenarios are considered with varying total sampling steps and subsample sizes (total sampling step, subsample size) = (6, 50), (3, 100), and (2, 150).

Subsample size may affect accuracy of the kernel smoothing estimation for the actively estimated weights. To this end, we examined the effects of different subset sizes at each sampling step on the estimation for model performance metrics. Given that the expected total of labeled data size is fixed at 300, we considered three scenarios with different combinations of total sampling steps and subsample sizes: (total sampling step, subsample size) $= (6, 50)$, (3, 100), and (2, 150). We focused on the AIIPW-Rec method, which showed superior performance compared to other methods in the previous results.

Figure 6 displays the average of the square root of MSE for the cross-entropy loss. Across the four datasets, the proposed method demonstrated stable estimation, regardless of the subsample size. The MSE for the Fashion-MNIST dataset is around 0.118 at subsample sizes 50 and 100, which is close to the MSE at subsample size 150 (MSE: 0.116). For the CIFAR-10 dataset, the MSE is 0.047 at subsample size 50, and the MSEs were nearly unchanged as the subsample size increased (0.043 at size 100 and 0.044 at size 150). For the Drug Consumption dataset, the MSE started with 0.077 at subsample size 50, and minor fluctuations were observed as the subsample size changed (0.075 at size 100 and 0.076 at size 150). Similarly, the NAFLD dataset had the MSE of 0.049 at size 50, with minor changes to 0.048 and back to 0.049 for sizes 100 and 150, respectively.

## Discussion

AT algorithms have been developed to evaluate pre-trained models without requiring extensive manual labeling of test data. This is accomplished by selecting more informative data points for labeling, thereby minimizing manual effort and additional costs. We extend existing AT frameworks with two novel estimators constructed on nonparametric smoothing estimation: AILUR and AIIPW. While the AILUR method requires retaining all historically estimated weights from the initial sampling step, the AIIPW method, in contrast, utilizes the calibrated weights corresponding to all labeled data as estimated in the most recent sampling step.

Our work demonstrates that both proposed estimators, AILUR and AIIPW, are more efficient than existing methods. Using MSE of the cross entropy as a metric for efficiency, we show that MSE of AIIPW was consistently lower than that of LUR across four distinct datasets and across sampling sizes. When considering different modeling strategies to update sampling probabilities, AIIPW that utilizes model recalibration outperformed AILUR and LUR in terms of efficiency as more labeled data was accumulated by the final sampling step. The LUR method employs adjusted inverse probability weightings (IPW) during sequential sampling steps. However, it is well known that estimators based on true sampling weights generally suffer from lower estimation efficiency when sampling probabilities are very low^13^. Moreover, we showed that the performance of the proposed estimators was robust to distinct real-world datasets and stable to variations in subsample size, number of sampling steps, performance metrics, and datasets for model evaluation.

Our research contributes to the literature on sample size determination in planning statistical studies^18^. In the numerical experiments, we assessed RE of AUC and AUPRC by comparing the estimation efficiency of the proposed methods relative to LUR (Fig. 5). Notably, both AIIPW and AILUR required fewer labeled data points to achieve MSE performance for AUC and AUPRC comparable to that of LUR. These results indicate that using AILUR and AIIPW may reduce the sample size requirements or sampling steps for model evaluation when compared to the standard AT framework, LUR.

Rigorous evaluation of machine learning and statistical models is critical for their safe use in real world settings. Our framework to evaluate the models in settings with incomplete data will enable the use of more reliable and accurate predictive models and increase confidence in analytic tools for decision making. Throughout this paper, we considered Poisson sampling to select subsets of data. However, other sampling schemes, such as sampling without replacement (SwR), are often used as an alternative^7,9^. Although both sampling schemes do not select a data point more than once over the sampling steps, sample sizes are fixed under the SwR scheme, while they are random under the Poisson sampling scheme. However, the SwR scheme requires the calculation of sampling probabilities for different categorical distributions corresponding to a subsample size at each sampling step. In contrast, for the Poisson sampling scheme, the sampling probability is calculated once within each sampling step to generate a subsample of an expected subsample size. We applied the proposed algorithm to the four datasets under the SwR scheme. The results were similar to those obtained under the Poisson sampling scheme (see Supplementary Fig. 1). The estimates were close to the benchmark, and the proposed AIIPW method outperformed the LUR method. Further comparative studies between these sampling schemes are left for future work.

An underlying assumption for AT is that test data comes from the same population as the training data. However, in practice, predictions generated by trained models may not be directly applicable to the test data due to factors such as covariate shift^19–21^. A framework of simultaneous active model modification and validation can be explored to revise original prediction models^22,23^ and provide suitable models to target populations, followed by evaluating the revised models.

### Supplementary Information

Supplementary Information.

## Data Availability

Fashion MNIST data, CIFAR-10 data, and Drug consumption data analysed during the current study are available at https://github.com/zalandoresearch/fashion-mnist, https://www.cs.toronto.edu/%7Ekriz/cifar.html, and https://archive.ics.uci.edu/dataset/373/drug+consumption+quantified, respectively. Non-alcoholic fatty liver disease data analysed during the current study is available from the corresponding author on reasonable request.
