# Supplementary material for: Towards optimal model evaluation: enhancing active testing with actively improved estimators
Source: Sci Rep. 2024 May 9;14:10690. doi: 10.1038/s41598-024-58633-3 (PMC11082224; doi:10.1038/s41598-024-58633-3)
Supplement: Supplementary file 1 — Supplementary Information. [file 41598_2024_58633_MOESM1_ESM.pdf]

Supplementary Information for "Towards Optimal Model Evaluation: Enhancing Active Testing with Actively Improved Estimators"

JooChul Lee*, Likhitha Kolla[1], and Jinbo Chen[2]

Department of Biostatistics, Epidemiology & Informatics, University of Pennsylvania, Perelman School of Medicine, Philadelphia, PA 19104, USA

*Corresponding author: joochul.lee@pennmedicine.upenn.edu
[1]Likhitha.Kolla@Pennmedicine.upenn.edu
[2]jinboche@pennmedicine.upenn.edu

Supplementary Note 1. Model re-calibration of multiclass data for alternative sampling probability

We first discuss kernel smoothing estimation for sampling weights for multiclass classification. Accuracy in multivariate kernel density estimation can deteriorate as the number of classes increases[1]. To address the 'curse of dimensionality', we estimate sampling weights using the kernel smoothing estimator

$$\hat{\mathbf{E}}\{\delta_i^s|f(\mathbf{X}_i)=z\} = \frac{\sum_{i=1}^N \delta_i^s K_b\{f(\mathbf{X}_i)-z\}}{\sum_{i=1}^N K_b\{f(\mathbf{X}_i)-z\}}$$

where $f(\mathbf{X}) \equiv \mathbf{E}[\mathcal{L}\{g(\mathbf{X}), Y\}|\mathbf{X}]$. Using the estimated weight, we conduct model re-calibration based on a multiclass logistic regression by considering $f(\mathbf{X})$ as the predictor. Let $\mathbf{Y}_i = (Y_{i,1}, ..., Y_{i,c})$ where $Y_{i,c} = 1$ if the outcome of the ith data point is the cth class for $c = 1, ..., C$. Let $\mathbf{h}\{f(\mathbf{X}_i), \boldsymbol{\theta}\} = [h_1\{f(\mathbf{X}_i), \boldsymbol{\theta}\}, ..., h_C\{f(\mathbf{X}_i), \boldsymbol{\theta}\}]$ where $\boldsymbol{\theta} = (\theta_1, ..., \theta_{C-1})$,

$$h_C\{f(\mathbf{X}_i), \boldsymbol{\theta}\} = \frac{1}{1+\sum_{m=1}^{C-1}\exp\{\theta_m f(\mathbf{X}_i)\}},$$

and

$$h_l\{f(\mathbf{X}_i), \boldsymbol{\theta}\} = \frac{\exp\{\theta_l f(\mathbf{X}_i)\}}{1+\sum_{m=1}^{C-1}\exp\{\theta_m f(\mathbf{X}_i)\}},$$

for $l = 1, ..., C-1$. We propose the model re-calibration for multiclass classification with the labeled data $\{\delta_i^s, \delta_i^s Y_i, \mathbf{X}_i, i = 1, ..., N\}$ using the estimating equation

$$\sum_{i=1}^N \frac{\delta_i^s f(\mathbf{X}_i)[\mathbf{Y}_i - \mathbf{h}\{f(\mathbf{X}_i), \boldsymbol{\theta}\}]}{\hat{\mathbf{E}}\{\delta_i^s|f(\mathbf{X}_i)\}} = \mathbf{0}. \tag{1}$$

Let $\mathbf{h}\{f(\mathbf{X}_i), \hat{\boldsymbol{\theta}}_s\}$ be the re-calibrated model where $\hat{\boldsymbol{\theta}}_s$ is the solution of the equation (1). We update the sampling probabilities using the re-calibrated model $\mathbf{h}\{f(\mathbf{X}_i), \hat{\boldsymbol{\theta}}_s\}$.

Supplementary Note 2. Detailed explanation of experiments

Updating methods for sampling probability

We first provide details of three updating approaches for the sampling probability: the original model to be assessed (Ori), random forest models (RF), and the proposed re-calibrated models (Rec). For the Ori method, we define the sampling probability as $P(\delta^s = 1|\mathbf{X}) \propto \sum_{i=0}^{c-1} g_c(\mathbf{X}) \log g_c(\mathbf{X})$. Let $g_c^{RF,s-1}(\mathbf{X})$ denote the random forest model constructed using the cumulative labeled data up to the $(s-1)^{th}$ sampling step. For the RF method, the sampling probability at the sth sampling step is defined as $P(\delta^s = 1|\mathbf{X}) \propto \sum_{i=0}^{c-1} g_c^{RF,s-1}(\mathbf{X}) \log g_c(\mathbf{X})$. For the Rec method, it is given by $P(\delta^s = 1|\mathbf{X}) \propto \sum_{i=0}^{c-1} h_c\{\hat{\theta}^{s-1} f(\mathbf{X}_i)\} \log g_c(\mathbf{X})$ where $h_c(\hat{\theta}^{s-1} f(\mathbf{X}_i))$ is the re-calibrated model obtained from the estimating equation in the equation (1).

Kernel smoothing estimation for weights

We considered Gaussian kernel smoothing estimator for sampling weights with different bandwidths: $b = N^{-0.05}$ for the AIILUR-RF method and $b = N^{-0.001}$ for the AIIPW-Ori and AIIPW-REC methods on the Fashion-MNIST data, $b = N^{-0.2}$ for AIILUR-RF and $b = N^{-0.35}$ for the AIIPW-Ori and AIIPW-REC methods on the CIFAR-10 data, $b = N^{-0.01}$ for the AIILUR-RF, AIIPW-Ori, and AIIPW-Rec methods on the Drug data, and $b = N^{-0.15}$ for AIILUR-RF and $b = N^{-0.25}$ for the AIIPW-Ori and AIIPW-REC methods on the NAFLD data. Here, N represents the total size of the test data.

Supplementary Note 3. Additional experiments

We applied the proposed methods to estimate performance metrics under sampling without replacement (SwR). Using the alternative sampling function based on Rec, we considered AIIPW and LUR estimators for the cross-entropy loss. We considered five sampling steps, a subsample size of 100 at each step, and 1000 repetitions. Estimates from the full test data were set as the benchmark metrics for comparison.

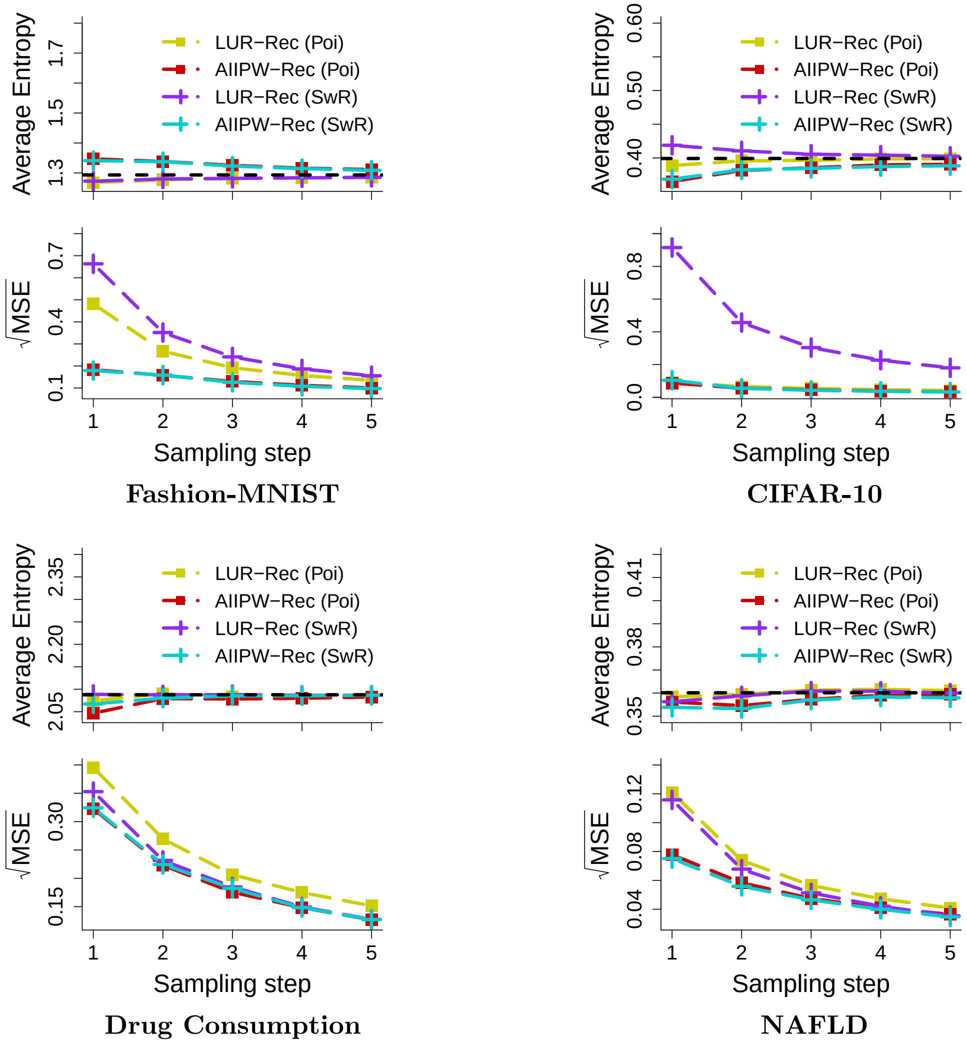

Figure S.1: Average of the cross-entropy loss and average of the square root of the mean squared error (MSE) using the Fashion-MNIST, CIFAR-10, and Drug Consumption datasets. LUR and AIIPW refer to the levelled unbiased risk estimator and the actively improved inverse probability weighting estimator, respectively. Rec denotes the use of the proposed re-calibrated models when updating the sampling probability. Poi and SwR indicate Poisson sampling and Sampling without replacemet, respectively.

Supplementary Reference

1. Scott, D. W. Feasibility of multivariate density estimates.. *Biometrika* **78,** 197–205 (1991).
